# Multiple positive solutions to a coupled systems of nonlinear fractional differential equations

**DOI:** 10.1186/s40064-016-2656-9

**Published:** 2016-07-19

**Authors:** Kamal Shah, Rahmat Ali Khan

**Affiliations:** Department of Mathematics, University of Malakand, Dir(L), Chakdara, Khyber Pakhtunkhwa Pakistan

**Keywords:** Fractional differential equations, Coupled system, Boundary condition, Generalized metric space, Contractions mapping, 34A08, 34B37, 35R11

## Abstract

In this article, we study existence, uniqueness and nonexistence of positive solution to a highly nonlinear coupled system of fractional order differential equations. Necessary and sufficient conditions for the existence and uniqueness of positive solution are developed by using Perov’s fixed point theorem for the considered problem. Further, we also established sufficient conditions for existence of multiplicity results for positive solutions. Also, we developed some conditions under which the considered coupled system of fractional order differential equations has no positive solution. Appropriate examples are also provided which demonstrate our results.

## Background

In recent years it is mainly proved that fractional differential equations are the best tools in the mathematical modeling of many phenomena in various field of physics, electrochemistry, viscoelasticity, control theory, image and signal processing etc, (see Hilfer 2000; Kilbas et al. 2006; Lakshmikantham et al. 2009; Podlubny 1999; Rossikhin and Shitikova 1997). Also, fractional differential equations are used in the modeling of various phenomena such as nonlinear oscillation of earthquake, Nutting’s law of charge transport in amorphous semi conductors, fluid dynamics, traffic model and non-Markorian diffusion process with memory. For the aforesaid application, see Mainardi (1995), Metzler and Klafer (2000), Scher and Montroll (1975), Wang et al. (2011). Moreover, considerable attention has been given to study mathematical epidemiology in term of fractional order models, this is due to the fact that fractional order models of epidemic disease are more realistic and provide great information as compared to classical order models. In last few years fractals and Chaos are also studied by using the tools of fractional differential equations. Besides from the above discussion, fractional order differential equations have also many applications in the fields of aerodynamics, fluid dynamics, physical chemistry, economics, polymerrheology, regular variation in thermodynamics, biophysics, blood flow phenomena, etc. Due to the important applications and uses of fractional differential equations, researchers of various field of mathematics, engineering, physics and computer science etc, gave much attention to study fractional differential equations. Another important application of fractional calculus has been found in condensed matter physics, where the fractional quantum Hall effect is one of the most attracting phenomena. For the present or planned technologies fractional order models are used by the implementation of an optical lattice setup. For detail, see Nielsen et al. (2013), Hagerstrom et al. (2012) and the reference there in. In networking systems it has been proved that several real networks in their degree of distribution obey a power-law. The presence of highly connected nodes in a scale-free network causes well known robustness against random failures. But on the other hand suffers from vulnerability to malicious attacks at their highly connected nodes. Fractional order models provide more realistic and accurate approach as compared to classical order models to study the afore said phenonmena, see Shang (2014). In last few years, the study of existence and uniqueness of solutions to boundary value problems for fractional order differential equations got much attention from many researchers and a number of research articles are available in the literature, we refer few of them in Deren (2015), Li et al. (2010), Khan and Shah (2015) and the reference therein. The iterative solutions to boundary and initial values problems of nonlinear fractional order differential equations were also studied by some authors (see Ahmad and Nieto 2008; Cui and Zou 2014; Shah et al. 2016 and the references therein). Moreover, existence and multiplicity of positive solutions to nonlinear boundary values problem of fractional order differential equations have been studied by many authors by using classical fixed point theorems, for example see Ahmad and Nieto (2009b), Bai (2008), Cui et al. (2012), Xu et al. (2009). Bai and Lü (2005), have studied the existence of multiple solutions for the following boundary value problems

$$\begin{aligned} \left\{ \begin{aligned}&{{\mathcal {D}}}^\alpha u(t) +f(t, u(t))=0, \quad t \in (0,1),\ 1<\alpha \le 2,\\&u(0)=u(1)=0. \end{aligned} \right. \end{aligned}$$

where ${{\mathcal {D}}}^\alpha$ is the standard Riemann–Liouville fractional derivative of order $\alpha ,$ and $f:[0,1]\times [0,\infty )\rightarrow [0,\infty )$ is continuous function. By means of classical fixed point theorems sufficient conditions were obtained for multiplicity of solutions. Recently, Goodrich (2010), considered the following class of nonlinear fractional differential equations with the given boundary conditions for multiplicity of positive solutions as

$$\begin{aligned} \left\{ \begin{aligned}&{{\mathcal {D}}}^\alpha u(t) +f(t, u(t))=0, \quad t \in (0,1),\ n-1<\alpha \le n,\\ &u^{(i)}(0)=0,\quad 0\le i\le n-2,\ \text {and}\ {{\mathcal {D}}}^{\delta } u(1)=0, \quad 2\le \delta \le n-2. \end{aligned} \right. \end{aligned}$$

where $n>3$ and $f:[0,1]\times [0,\infty )\rightarrow [0,\infty )$ is continuous function. Many problems in applied sciences can be modeled as coupled system of differential equations with different type of boundary conditions. The coupled system of fractional order differential equations have many application in computer networking, see Li et al. (2015a, b), Suo et al. (2013). Boundary values problems for coupled systems with ordinary derivatives are well studied, however, coupled systems with fractional derivatives have attracted the attention quite recently. Most of the biological, physical, computer network model and chemical models etc, are in the form of coupled system (see Anastassiou et al. 2011; Chasnov 2009; Lia et al. 2015). Due to these important applications and uses of coupled systems of fractional order differential equations, considerable attention was given to study coupled system for the existence, uniqueness and multiplicity of positive solutions, for detail we refer Miller and Ross (1993), Shah and Khan (2015), Su (2009), Shah et al. (2015) and the references therein. As Bai and Feng (2004), established sufficient conditions for existence of positive solution to a coupled system of fractional differential equations as given by the

$$\begin{aligned} \left\{ \begin{aligned}&{{\mathcal{D}}}^{\alpha }u(t) = f(t, v(t));\quad 0<t<1,\\& {{\mathcal {D}}}^{\beta }v(t) = g(t, u(t));\quad 0<t<1, \end{aligned}\right. \end{aligned}$$

where $1<\alpha , \beta < 1,\; f,g:[0,1]\times R \rightarrow R$ are nonlinear continuous functions. Wang et al. (2010), developed sufficient conditions for existence and uniqueness of solution for the coupled system with three point boundary conditions of the form

$$\begin{aligned} \left\{ \begin{aligned}&{{\mathcal {D}}}^\alpha u(t) = f(t, v(t)),\, {{\mathcal {D}}}^{\beta }v(t) = g(t, u(t)); \quad 0<t<1,\\ &u(0)=v(0) =0;\ u(1) = au(\xi ),\ v(1) =bv(\xi ), \end{aligned}\right. \end{aligned}$$

where $1<\alpha , \beta< 2,\ 0\le a,b\le 1, 0<\xi <1,$ and $f,g:[0,1]\times [0,\infty ) \rightarrow [0,\infty )$ are nonlinear continuous functions. Rehman and Khan (2010), established sufficient conditions for multiplicity results for positive solutions to the following coupled system of nonlinear boundary value problem of fractional differential equations as given by

$$\begin{aligned} \left\{ \begin{aligned}&{{\mathcal {D}}}^{\alpha }u(t) + \lambda a(t)f(v(t))=0;\ {{\mathcal {D}}}^{\beta }v(t)+ \lambda b(t)g(u(t))=0; \quad 0<t<1,\\ &u(0) =u^{\prime }(0)=u^{\prime \prime }(0)=\cdots =u^{(n-2)}(0)=u^{(n-1)}(0)=0; \quad u(1)=0,\\ &v(0) =v^{\prime }(0)=v^{\prime \prime }(0)=\cdots =v^{(n-2)}(0)=v^{(n-1)}(0)=0;\quad v(1)=0, \end{aligned} \right. \end{aligned}$$

where $n-1<\alpha , \beta \le n, \lambda >0,\ f,g:[0,\infty ) \rightarrow [0,\infty )$ are continuous. Jalili and Samet (2014), studied existence and uniqueness as well as multiplicity of positive solutions to the following coupled system of boundary value problems of fractional differential equations

$$\begin{aligned} \left\{ \begin{aligned}&{{\mathcal {D}}}^{\alpha }u(t) + f(t, u(t), v(t))=0;\quad t\in (0,1);\ n-1<\alpha \le n,\\ &{{\mathcal {D}}}^{\beta }v(t) + g(t, u(t), v(t))=0;\quad t\in (0,1); \ n-1<\beta \le n,\\ &u^{(i)}(0)=v^{(i)}(0)=0,\quad i=0,1,2\ldots n-2,\\&{{\mathcal {D}}}^{\gamma } u(1) ={{\mathcal {D}}}^{\delta } v(1)= 0, \end{aligned} \right. \end{aligned}$$

where $2<\gamma ,\ \delta \le n-2,\ n>3, \;f,g:I \times [0,\infty ) \times [0,\infty ) \rightarrow [0,\infty )$ are continuous.

The aim of this paper is to study the existence, uniqueness as well as non-existence conditions for positive solution to the following system of non-linear fractional order differential equations with four point boundary conditions

1 $$\begin{aligned} \left\{ \begin{aligned}&{{\mathcal {D}}}^{\alpha } u(t)+\phi (t,u(t),v(t))=0; \quad t\in (0,1);\ n-1<\alpha \le n,\\&{{\mathcal {D}}}^{\beta } v(t)+\psi (t,u(t),v(t))=0; \quad t\in (0,1);\ n-1<\beta \le n,\\&u(0)= u^{\prime }(0)=u^{\prime \prime }(0)=\cdots =u^{(n-2)}(0)=0; \quad {{\mathcal {D}}}^{\delta }u(1) = \lambda {{\mathcal {D}}}^{\delta }u(\eta ),\\&v(0)= v^{\prime }(0)=v^{\prime \prime }(0)=\cdots =v^{(n-2)}(0)=0; \quad {{\mathcal {D}}}^{\gamma }v(1) = \mu {{\mathcal {D}}}^{\gamma }v(\xi ). \end{aligned} \right. \end{aligned}$$

where $n>3,\ \alpha - \delta \ge 1, \beta - \gamma \ge 1$ and $0<\delta , \gamma \le n-2,\ \lambda ,\mu \in (0,\infty ),\ 0<\eta ,\xi <1 , \phi ,\,\psi :[0,1]\times [0,\infty )\times [0,\infty ) \rightarrow [0,\infty )$ are continuous functions and ${{\mathcal {D}}}^\alpha ,\ {{\mathcal {D}}}^\beta$ stand for Riemann–Liouville fractional derivative of order $\alpha ,\beta$ respectively. Sufficient conditions are developed for uniqueness of solution of system (1), by using Perov’s fixed theorem. Moreover by means of some classical fixed point theorems of cone type, we develop necessary and sufficient conditions under which the considered system has at least one , two or more positive solutions. Also, we develop conditions for nonexistence of positive solution for system (1). We also provide some examples to illustrate our main results.

## Preliminaries

To proceed further, we recall some basic definitions and well known results of functional analysis, fixed point theory and fractional calculus (see i.e. Agarwal et al. 2004; Deimling 1985; Jalili and Samet 2014; Krasnoselskii 1964; Miller and Ross 1993; Podlubny 1993; Szilárd 2003; Zeidler 1986).

### **Definition 1**

The fractional integral of order $\alpha >0$ of a function $y:(0,\infty )\rightarrow {{\mathbb {R}}}$ is defined by

$$\begin{aligned} {{\mathcal {I}}}^{\alpha }y(t)= \frac{1}{\Gamma (\alpha )}\int _{0}^{t}(t-s)^{\alpha -1}y(s)ds, \end{aligned}$$

provided the integral is pointwise defined on $(0,\infty )$.

### **Definition 2**

The Riemann–Liouville fractional derivative of order $\alpha >0$ of a function $y\in C^{n}[0,1]$ is defined by

$$\begin{aligned} {{\mathcal {D}}}_{0+}^{\alpha }y(t)=\frac{1}{\Gamma (n-\alpha )}\left( \frac{d}{dt}\right) ^{n} \int _{0}^{t}(t-s)^{n-\alpha -1}y(s)ds, \end{aligned}$$

where $n=\lceil \alpha \rceil +1, \lceil \alpha \rceil$ represents integer part of $\alpha$.

The following results need in the sequel,

### **Lemma 3**

Ahmad and Nieto (2009a, b) *Let*$\alpha > 0$*then for arbitrary*$C_i \in R,\; i=0,1,2,\ldots ,n,\;n=[\alpha ]+1,$*we have*

$$\begin{aligned} {{\mathcal {I}}}^{\alpha }{{\mathcal {D}}}^{\alpha }y(t) =y(t)+C_1 t^{\alpha -1}+C_2t^{\alpha -2}+\ldots +C_{n}t^{\alpha -n}. \end{aligned}$$

### **Lemma 4**

Agarwal et al. (2004) *LetXbe a Banach space with*$P \subseteq X$*closed and convex. LetUbe a relatively open subset ofPwith*$0 \in U$*and*${{\mathcal {A}}}:\overline{U}\rightarrow U$*be a continuous and compact(completely continuous) mapping. Then either*

1. *The mapping*${{\mathcal {A}}}$*has a fixed point in*$\overline{U}$*or*
2. *There exist*$u\in \partial U$*and*$\lambda \in (0,1)$*with*$u=\lambda {{\mathcal {A}}}u.$

### **Lemma 5**

Podlubny (1993) *LetPbe a cone of real Banach spaceXand let*$\Omega _1$*and*$\Omega _2$*be two bounded open sets inXsuch that*$0\in \Omega _1 \subset \overline{\Omega }_1 \subset \Omega _2$. *Let operator*${{\mathcal {A}}}:P\cap (\overline{\Omega }_2 \setminus \Omega _1)\rightarrow P$*be completely continuous operator. If one of the following two conditions holds:*

1. $\Vert {{\mathcal {A}}}u\Vert \le \Vert u\Vert$*for all*$u\in P \cap \partial \Omega _1;\;\Vert {{\mathcal {A}}}u\Vert \ge \Vert u\Vert$, *for all*$u\in P \cap \partial \Omega _2;$
2. $\Vert {{\mathcal {A}}}u\Vert \ge \Vert u\Vert$*for all*$u \in P \cap \partial \Omega _1;\;\Vert {{\mathcal {A}}}u\Vert \le \Vert u\Vert$, *for all*$u\in P \cap \partial \Omega _2$.

Then ${{\mathcal {A}}}$ has at least one fixed point in $P\cap (\overline{\Omega }_2 \setminus \Omega _1).$

### **Definition 6**

Jalili and Samet (2014) For a nonempty set *X*, the mapping $d:X \times X \rightarrow R^n$ is called a generalized metric on *X* if $\text {for all}\ x,y,\ \text {and}\ u,v \in X$ with $v\ne x,v\ne u,v \ne y$ satisfies

$$M_1.$$
$$d(u,v)=0_{R^n}\ \text {if and only if}\ u=v,\ \text {for all}\ u,v \in X;$$
$$M_2.$$
$$d(u,v)=d(v,u),\ \text {for all}\ u,v \in X;$$ (symmetric property)
$$M_3.$$
$$d(x,y) \le d(x,v)+d(v,u)+d(u,y),\ \text {for all}\ x,y, u,v, \in X$$ (tetrahedral inequality).

Moreover the pairs (*X*, *d*) is called generalized metric space.

### **Definition 7**

Jalili and Samet (2014) Let $M=\{M_{n,n} \in R_{+}^{n\times n}\}$, the system of all $n\times n$ matrices with positive element. For any matrix *A* the spectral radius is defined by $\rho (A)=\sup \{|\lambda _i|,i=1,2,\ldots ,n\}$, where $\lambda _i,(i=1,2,\ldots ,n)$ are the eigenvalues of the matrix *A*.

### **Lemma 8**

Jalili and Samet (2014) *Let* (*M*, *d*) *be a complete generalized metric space and let*${{\mathcal {A}}}:M \rightarrow M$*be an operator such that there exists a matrix*$A\in M$*with*

$$\begin{aligned} d({{\mathcal {A}}}u,{{\mathcal {A}}}v) \le A d(u,v), \text {for all}\ u,v \in M. \end{aligned}$$

*if*$\rho (A) <1$, *then*${{\mathcal {A}}}$*has a fixed point*$w \in M$. *Further, for any*$u_0$*the iterative sequence*$u_{n+1}={{\mathcal {A}}}u_n$*converges tow*.

## Main results

This section is concerned to the existence, uniqueness and multiplicity results of positive solutions for boundary value problem (1). We begin with the following lemma.

### **Lemma 9**

*Let*$y \in C[0,1]$*then the boundary value problem*

2 $$\begin{aligned} \begin{aligned}&{{\mathcal {D}}}^{\alpha }u(t) + y(t) = 0;\;\; 0<t<1; n-1<\alpha \le n,\\&u(0)=u^{\prime }(0)=\cdots =u^{(n-2)}(0)=0;~ {{\mathcal {D}}}^\delta u(1) = \lambda {{\mathcal {D}}}^\delta u(\eta ), \end{aligned} \end{aligned}$$

*where*$0<\eta<1,\ \lambda \in (0,\infty ), \;\;0<\delta \le n-2,$*has a unique positive solution given by*

$$\begin{aligned} u(t) = \int \limits _0^1 {{\mathcal {G}}}_\alpha (t,s)y(s)ds, \end{aligned}$$

*where*${{\mathcal {G}}}_\alpha (t,s)$*is a Green’s function given by*

3 $$\begin{aligned}&{{\mathcal {G}}}_\alpha (t,s)\nonumber \\&\quad = \frac{1}{\Gamma (\alpha )}\left\{ \begin{array}{ll} d_1 t^{\alpha -1}(1-s)^{\alpha -\delta -1}-(t-s)^{\alpha -1} -d_1\lambda t^{\alpha -1}(\eta -s)^{\alpha -\delta -1};& 0 \le s \le t\le \eta \le 1,\\ d_1 t^{\alpha -1}(1-s)^{\alpha -\delta -1}-(t-s)^{\alpha -1};& 0 \le \eta \le s \le t \le 1,\\ d_1t^{\alpha -1}(1-s)^{\alpha -\delta -1}-d_1t^{\alpha -1}\lambda (\eta -s)^{\alpha -\delta -1};& 0 \le t \le \eta \le s \le 1,\\ d_1t^{\alpha -1}(1-s)^{\alpha -\delta -1};& 0 \le \eta \le t \le s \le 1, \end{array} \right. \end{aligned}$$

*where*$$d_1=\frac{1}{1-\lambda \eta ^{\alpha -\delta -1}}.$$

### *Proof*

By applying ${{\mathcal {I}}}^\alpha$ and using Lemma 3, the general solution of linear boundary value problem (2) is given by

4 $$\begin{aligned} u(t) = -{{\mathcal {I}}}^\alpha y(t)+c_1t^{\alpha -1}+c_2t^{\alpha -2}+\cdots +c_nt^{\alpha -n}. \end{aligned}$$

With the help of boundary and initial conditions of Eq. (2) and ${{\mathcal {D}}}^\delta [t^{\alpha -1}]= \frac{\Gamma (\alpha )}{\Gamma (\alpha -\delta )}t^{\alpha -\delta -1}$, we get $c_2=c_3=\cdots =c_n=0$ and using $d_1= \frac{1}{1-\lambda \eta ^{\alpha -\delta -1}},$ we have

$$\begin{aligned} c_1=\frac{d_1}{\Gamma (\alpha )}\left[ \int _0^1 (1-s)^{\alpha -\delta -1}y(s)ds-\lambda \int _0^\eta ( \eta -s)^{\alpha -\delta -1}y(s)ds \right] \end{aligned}$$

and thus (4) becomes

$$\begin{aligned} \begin{aligned} u(t)&=-{{\mathcal {I}}}^\alpha y(t)+\frac{d_1 t^{\alpha -1}}{\Gamma (\alpha )}\left[ \int _0^1(1-s)^{\alpha -\delta -1}y(s)ds-\lambda \int _0^\eta (\eta -s)^{\alpha -\delta -1}y(s)ds \right] \\&=-\frac{1}{\Gamma (\alpha )}\int _0^t(t-s)^{\alpha -1}y(s)ds\\&\quad +\frac{d_1 t^{\alpha -1}}{\Gamma (\alpha )}\left[ \int _0^1(1-s)^{\alpha -\delta -1}y(s)ds -\lambda \int _0^\eta (\eta -s)^{\alpha -\delta -1}y(s)ds \right] \\&=\int _0^1 {{\mathcal {G}}}_\alpha (t,s)y(s)ds, \end{aligned} \end{aligned}$$

where ${{\mathcal {G}}}_\alpha (t,s)$ is the Green’s function of linear boundary value problem (2). Similarly, we can obtain $v(t)=\int _0^1 {{\mathcal {G}}}_\beta (t,s)z(s)ds,$ where ${{\mathcal {G}}}_\beta (t,s)$ is the Green’s function for the second equation of the system (1) and given by

5 $$\begin{aligned} {{\mathcal {G}}}_\beta (t,s)= \frac{1}{\Gamma (\beta )}\left\{ \begin{array}{ll} d_2 t^{\beta -1}(1-s)^{\beta -\gamma -1}-(t-s)^{\beta -1}-d_2\mu t^{\beta -1}(\xi -s)^{\beta -\gamma -1};& 0 \le s \le t\le \xi \le 1,\\ d_2 t^{\beta -1}(1-s)^{\beta -\gamma -1}-(t-s)^{\beta -1};& 0 \le \xi \le s \le t \le 1,\\ d_2t^{\beta -1}(1-s)^{\beta -\gamma -1}-d_2t^{\beta -1}\mu (\xi -s)^{\beta -\gamma -1};& 0 \le t \le \xi \le s \le 1,\\ d_2t^{\beta -1}(1-s)^{\beta -\gamma -1}; &0 \le \xi \le t \le s \le 1, \end{array} \right. \end{aligned}$$

where $$d_2=\frac{1}{1-\mu \xi ^{\beta -\gamma -1}}.$$$$\square$$

We recall the following lemma found in Jalili and Samet (2014) and Yang (2012). The proof is omitted because the proof is like the proof given in Goodrich (2010).

### **Lemma 10**

*Let*$G(t,s)=({{\mathcal {G}}}_\alpha (t,s),~{{\mathcal {G}}}_\beta (t,s))$*be the Green’s function of* (1). *This Green’s functionG*(*t*, *s*) *has some properties given by*

$$(P_1)$$
*G*(*t*, *s*) *is continuous function on the unit square for all*$(t,s)\in [0,1]\times [0,1];$
$$(P_2)$$
$G(t,s)\ge 0$*for all*$t,s \in [0,1]$*and*$G(t,s) > 0$*for all*$t,s \in (0,1);$
$$(P_3)$$
$${\displaystyle \max _{0 \le t \le 1}} G(t,s) = G(1,s), \forall \ s \in [0,1];$$
$$(P_4)$$
$${\displaystyle \min _{t\in [\omega ,1-\omega ] }} G(t,s) \ge \gamma (s)G(1,s)$$*for each*$$\omega \in (0,\frac{1}{2}),\ s\in (0,1),$$

*where*$$\gamma = \min \{\gamma _\alpha =\omega ^{\alpha -1}, \gamma _\beta =\omega ^{\beta -1}\}.$$

Further we define some fundamental results which will be used throughout in this paper. Let us define $X=\{u(t)|u \in C[0,1]\}$ endowed with the norm $||u||=\max _{t\in [0,1]}|u(t)|.$ Further the norm for the product space we define as $||(u,v)||=||u||+||v||.$ Obviously $(X\times X,||\cdot ||)$ is a Banach space.

Let $J=[\omega ,1-\omega ]$, then, we define the cone $P\subset X\times X$ by

$$\begin{aligned} \begin{aligned} P&=\{(u,v) \in X\times X: \min _{t \in J}[u(t)+v(t)]\ge \gamma ||(u,v)||\}.\\ P_r&= \{(u,v)\in P: \Vert (u,v)\Vert \le r\}, \,\partial P_r = \{(u,v)\in P:\Vert (u,v)\Vert =r\}. \end{aligned} \end{aligned}$$

Now inview of Lemma 9, we can write system (1) as an equivalent coupled system of integral equations given as

6 $$\begin{aligned} \left\{ \begin{aligned} u(t)&= \int \limits _0^1{{\mathcal {G}}}_\alpha (t,s)\phi (s,u(s),v(s))ds, \\ v(t)&= \int \limits _0^1{{\mathcal {G}}}_\beta (t,s)\psi (s,u(s),v(s))ds. \end{aligned}\right. \end{aligned}$$

Let ${{\mathcal {A}}}:X\times X \rightarrow X \times X$ be the operator defined as

7 $$\begin{aligned} \begin{array}{ll} {{\mathcal {A}}}(u,v)(t) & = \left( \int \limits _0^1 {{\mathcal {G}}}_\alpha (t,s) \phi (s, u(s),v(s))ds, \;\;\int \limits _0^1{{\mathcal {G}}}_\beta (t,s) \psi (s, u(s), v(s))ds \right) \\ & = \bigg ({{\mathcal {A}}}_1(u, v)(t),{{\mathcal {A}}}_2(u, v)(t)\bigg ). \end{array}. \end{aligned}$$

Then the fixed point of operator ${{\mathcal {A}}}$ coincides with the solution of coupled system (1).

### **Theorem 11**

*Assume that*$f,g:[0,1]\times [0,\infty )\times [0,\infty )\rightarrow [0,\infty )$*are continuous. Then*${{\mathcal {A}}}(P)\subset P \ \text {and} \ {{\mathcal {A}}}:P\rightarrow P$*is completely continuous, where*${{\mathcal {A}}}$*is defined in* (7).

### *Proof*

To derive ${{\mathcal {A}}}(P)\subset P$, let $(u,v) \in P$,then by Lemma 10, we have ${{\mathcal {A}}}(u,v) \in P$. Further from property $(P_4)$ and for all $t\in J$, we get

8 $$\begin{aligned} {{\mathcal {A}}}_1 (u(t),v(t)) =\int \limits _0^1\mathcal { G}_\alpha (t,s)\phi (s, u(s),v(s))ds\ge \gamma _\alpha \int \limits _0^1 {{\mathcal {G}}}_\alpha (1,s)\phi (s,u(s)),v(s)ds. \end{aligned}$$

Also from $(P_3)$, we obtain

9 $$\begin{aligned} {{\mathcal {A}}}_1 (u(t),v(t)) =\int \limits _0^1{{\mathcal {G}}}_\alpha (t,s)\phi (s, u(s),v(s))ds\le \int \limits _0^1 {{\mathcal {G}}}_\alpha (1,s)\phi (s,u(s)),v(s)ds. \end{aligned}$$

Thus from (8) and (9), we have

$$\begin{aligned} {{\mathcal {A}}}_1(u(t),v(t))\ge \gamma \Vert {{\mathcal {A}}}_1(u,v)\Vert ,\ \text {for all} \ t\in J. \end{aligned}$$

Similarly, one can write that

$$\begin{aligned}&{{\mathcal {A}}}_2(u(t),v(t)) \ge \gamma \Vert {{\mathcal {A}}}_2(u,v)\Vert , \ \text {for all} \ t\in J.\\&\text {Thus}\ {{\mathcal {A}}}_1(u(t),v(t))+{{\mathcal {A}}}_2(u(t),v(t))\ge \gamma \Vert {{\mathcal {A}}}(u,v)\Vert , \ \text {for all} \ t\in J,\\&\min _{t\in J}[{{\mathcal {A}}}_1(u(t),v(t))+{{\mathcal {A}}}_2(u(t),v(t))]\ge \gamma \Vert {{\mathcal {A}}}(u,v)\Vert . \end{aligned}$$

Hence we have ${{\mathcal {A}}}(u,v) \in P\Rightarrow {{\mathcal {A}}}(P)\subset P.$ Next by similar proof of Theorem 1 of Shah and Khan (2015) and using Arzel$\acute{a}$-Ascoli’s theorem, one can easily prove that ${{\mathcal {A}}}:P\rightarrow P$ is completely continuous. $\square$

### **Theorem 12**

*Assume that*$\phi$*and*$\psi$*are continuous on*$[0,1]\times [0,\infty )\times (0,\infty )\rightarrow [0,\infty )$, *and there exist*$f_i(t),g_i(t),(i=1,2):(0,1)\rightarrow [0,\infty )$*that satisfy*

$$(A_1)$$
$$|\phi (t,u,v)-\phi (t,\bar{u},\bar{v})| \le f_1(t)|u-\bar{u}|+g_1(t)|v-\bar{v}|,$$*for*$$t\in (0,1)$$*and*$$u,v,\bar{u},\bar{v} \ge 0;$$
$$(A_2)$$
$$|\psi (t,u, v)-\psi (t,\bar{u},\bar{v})| \le f_2(t) |u-\bar{u}|+g_2(t)|v-\bar{v}|,$$*for*$$t \in (0,1)$$* and*$$u,u,\bar{u},\bar{v} \ge 0;$$
$$(A_3)$$
$\mathcal {\rho }(A)<1,$*where*$A\in \{M_{2,2}\in R^{2\times 2}_+\}$*is a matrix given by*
  $$\begin{aligned} \left[ \begin{aligned}&\int _0^1{{\mathcal {G}}}_\alpha (1,s)f_1(s)ds&\ \ \int _0^1{{\mathcal {G}}}_\alpha (1,s)g_1(s)ds \\&\int _0^1{{\mathcal {G}}}_\beta (1,s)f_2(s)ds&\ \ \int _0^1{{\mathcal {G}}}_\beta (1,s)g_2(s)ds \end{aligned} \right] . \end{aligned}$$

*Then the system* (1) *has a unique positive solution*$(u,v) \in P$.

### *Proof*

Let us define a generalized metric $d:X^2\times X^2\rightarrow R^2$ by

$$\begin{aligned}d((u,v),(\bar{u},\bar{v}))=\left( \begin{array}{c} ||u-\bar{u}|| \\ ||v-\bar{v}|| \\ \end{array} \right) ,\ \text {for all}\ (u,v),(\bar{u},\bar{v})\in X\times X. \end{aligned}$$

Obviously $(X\times X,d)$ is a generalized complete metric space. Then for any $(u,v),(\bar{u},\bar{v})\in X\times X$ and using property $(P_3)$ we get

$$\begin{aligned} |{{\mathcal {A}}}_1(u,v)(t)-{{\mathcal {A}}}_1(\bar{u},\bar{v})(t)|\le & \max _{t \in [0,1]} \int\limits _0^1 |G_\alpha (t,s)| [|\phi (s,u(s), v(s))-\phi (s, \bar{u}(s),\bar{v}(s))|]ds\\\le & \int\limits _0^1 {{\mathcal {G}}}_{\alpha }(1,s)[f_1(s)\Vert u-\bar{u}\Vert +g_1(s)\Vert v- \bar{v}\Vert ]ds\\\le & \int\limits _0^1f_1(s){{\mathcal {G}}}_{\alpha }(1,s)ds\Vert u-\bar{u}\Vert + \int\limits _0^1g_1(s){{\mathcal {G}}}_{\alpha }(1,s)ds\Vert v-\bar{v}\Vert .\\ \end{aligned}$$

Similarly we can show that

$$\begin{aligned} |{{\mathcal {A}}}_2(u,v)-{{\mathcal {A}}}_2(\bar{u},\bar{v})|\le & \int _0^1f_2(s){{\mathcal {G}}}_{\beta }(1,s)ds\Vert u-\bar{u}\Vert \\&+ \int _0^1g_2(s){{\mathcal {G}}}_{\beta }(1,s)ds\Vert v-\bar{v}\Vert . \end{aligned}$$

Thus we have

$$\begin{aligned} |{{\mathcal {A}}}(u,v)-{{\mathcal {A}}}(\bar{u},\bar{v})|\le A d\left( (u,v),(\bar{u},\bar{v})\right), \quad \forall (u,v),(\bar{u},\bar{v}) \in X\times X, \end{aligned}$$

where

$$\begin{aligned}A=\left[ \begin{aligned}&\int _0^1{{\mathcal {G}}}_\alpha (1,s)f_1(s)ds&\ \ \int _0^1{{\mathcal {G}}}_\alpha (1,s)g_1(s)ds \\&\int _0^1{{\mathcal {G}}}_\beta (1,s)f_2(s)ds&\ \ \int _0^1{{\mathcal {G}}}_\beta (1,s)g_2(s)ds \end{aligned} \right] . \end{aligned}$$

As $\mathcal {\rho }(A) <1,$ by means of Lemma 8, system (1) has a unique positive solution. $\square$

### **Theorem 13**

*Let*$\phi$*and*$\psi$*are continuous on*$[0,1]\times [0,\infty )\times (0,\infty )\rightarrow [0,\infty )$*and there exist*$c_i,d_i,e_i (i=1,2):(0,1)\rightarrow [0,\infty )$*satisfying:*

$$(A_4)$$
$$\phi (t, u(t),~v(t)) \le c_1(t)+d_1(t)u(t)+e_1(t)v(t),\ t\in (0,1), u,v \ge 0;$$
$$(A_5)$$
$$\psi (t, u(t),~v(t)) \le c_2(t)+d_2(t)u(t)+e_2(t)v(t),\ t\in (0,1), u,v \ge 0;$$
$$(A_6)$$
$$\Delta _1 = \int \limits _0^1 {{\mathcal {G}}}_\alpha (1,s)c_1(s)ds< \infty ,\;\Lambda _1 = \int \limits _0^1 {{\mathcal {G}}}_\alpha (1,s)[d_1(s)+e_1(s)]ds < \frac{1}{2};$$
$$(A_7)$$
$$\Delta _2 = \int \limits _0^1 {{\mathcal {G}}}_\beta (1,s)c_2(s)ds< \infty ,\;\Lambda _2 = \int \limits _0^1 G_\beta (s,s)[d_2(s)+e_2(s)]ds < \frac{1}{2}.$$
  *Then the system* (1) *has at least one positive solution in*

$$\begin{aligned} \bigg \{(u,v) \in P : \Vert (u,v)\Vert \le r\bigg \},\ {{where}}\ \max \bigg \{\dfrac{\Delta _1}{\frac{1}{2}-\Lambda _1}, \dfrac{\Delta _2}{\frac{1}{2}-\Lambda _2} \bigg \}<r. \end{aligned}$$

### *Proof*

Defined $$\Omega =\bigg \{(u,v) \in P : \Vert (u,v)\Vert < r \bigg \}$$ with $$\max \bigg \{\dfrac{\Delta _1}{\frac{1}{2}-\Lambda _1}, \dfrac{\Delta _2}{\frac{1}{2}-\Lambda _2}\bigg \}<r.$$

Also inview of Theorem 11, the operator ${{\mathcal {A}}}:\overline{\Omega } \rightarrow P$ is completely continuous. Let $(u, v)\in \Omega$, such that $\Vert (u, v)\Vert <r.$ Then, we have

$$\begin{aligned} \begin{aligned} \Vert {{\mathcal {A}}}_1(u,v)\Vert&=\max _{t\in [0, 1]}\bigg |\int _0^1{{\mathcal {G}}}_\alpha (t,s)\phi (s,u(s),v(s))\bigg |ds\\&\le \bigg (\int _0^1{{\mathcal {G}}}_\alpha (1,s)c_1(s)ds+\int _0^1{{\mathcal {G}}}_\alpha (1,s)d_1(s)|u(s)|ds+\int _0^1{{\mathcal {G}}}_\alpha (1,s)e_1(s)|v(s)|ds\bigg )\\&\le \int _0^1{{\mathcal {G}}}_\alpha (1,s)c_1(s)ds+r\bigg [\int _0^1{{\mathcal {G}}}_\alpha (1,s)[d_1(s)+e_1(s)]ds\bigg ]\\&\le \Delta _1+r\Lambda _1 < \frac{r}{2}, \end{aligned} \end{aligned}$$

similarly, $\Vert {{\mathcal {A}}}_2(u,v)\Vert < \frac{r}{2}$, thus $\Vert {{\mathcal {A}}}(u,v)\Vert < r$. Therefore, thank to Lemma 4, we have ${{\mathcal {A}}}(u,v)\in \overline{\Omega }.$ Therefore ${{\mathcal {A}}}:\overline{\Omega }\rightarrow \overline{\Omega }.$

Let there exist $\zeta \in (0, 1)$ and $(u,v) \in \partial \Omega$ such that $(u,v)=\zeta {{\mathcal {A}}}(u,v).$ Then inview of assumptions $(A_4), (A_5)$ and using Property $(P_4)$ of Lemma 10, we obtain for all $t \in [0,1]$

$$\begin{aligned} \begin{aligned} |u(t)|&\le \zeta \int _0^1{{\mathcal {G}}}_\alpha (t,s)|\phi (s,u(s),v(s))|ds\\&\quad \le \zeta \bigg (\int _0^1{{\mathcal {G}}}_\alpha (1,s)c_1(s)ds+\int _0^1{{\mathcal {G}}}_\alpha (1,s)d_1(s)u(s)ds+\int _0^1{{\mathcal {G}}}_\alpha (1,s)e_1(s)v(s)ds\bigg )\\&\quad \le \zeta \bigg (\Delta _1+r\Lambda _1\bigg )\\&\quad< \zeta \frac{r}{2}\\&\quad \text {which implies that}\ \Vert u\Vert < \zeta \frac{r}{2}, \end{aligned} \end{aligned}$$

similarly, one can show that $\Vert v\Vert < \zeta \frac{r}{2}.$

From which, we have $$\Vert (u,v)\Vert < \zeta r ,\ \text {with}\ \zeta \in (0, 1)$$ which is a contradiction that $$(u,v) \in \partial \Omega$$ as $$r=\Vert (u,v)\Vert .$$ Thus by mean of Lemma 4, $${{\mathcal {A}}}$$ has at least one fixed point $$(u, v)\in \overline{\Omega }.$$$$\square$$

Next we use the following assumptions and notations:

$$(C_1)$$
$\phi ,\psi :[0,1]\times [0,\infty )\times [0,\infty ) \rightarrow [0,\infty )$ are continuous and $\phi (t,0,0)=\psi (t,0,0)=0$ uniformly with respect to *t* on [0, 1].
$$(C_2)$$
${{\mathcal {G}}}_\alpha (1,s), {{\mathcal {G}}}_\beta (1,s)$ defined in Lemma 10 satisfy
  $$\begin{aligned} 0< \int \limits _0^1 {{\mathcal {G}}}_\alpha (1,s)ds<\infty ,\ 0< \int \limits _0^1 {{\mathcal {G}}}_\beta (1,s)ds<\infty ; \end{aligned}$$
$$(C_3)$$
Let these limit hold
  $$\begin{aligned} \begin{aligned} \phi ^\sigma&= \lim _{(u,v)\rightarrow (\sigma ,\sigma )}\sup _{t\in [0,1]} \dfrac{\phi (t,u,v)}{u+v}, \; \psi ^\sigma = \lim _{(u,v)\rightarrow (\sigma ,\sigma )}\sup _{t\in [0,1]} \dfrac{\psi (t,u,v)}{u+v},\\ \phi _\sigma&= \lim _{(u,v)\rightarrow (\sigma ,\sigma )}\inf _{t\in [0,1]} \dfrac{\phi (t,u,v)}{u+v}, \; \psi _\sigma = \lim _{(u,v)\rightarrow (\sigma ,\sigma )}\inf _{t\in [0,1]} \dfrac{\psi (t,u,v)}{u+v},\ \text {where}\ \sigma \in \{0,\infty \}. \end{aligned} \end{aligned}$$

$$\begin{aligned} \begin{aligned} \sigma _\alpha = \max _{t\in [0,1]} \int \limits _0^1 {{\mathcal {G}}}_\alpha (t,s)ds ,\;\sigma _\beta =\max _ {t\in [0,1]}\int \limits _0^1 {{\mathcal {G}}}_{\beta }(t,s)ds. \end{aligned} \end{aligned}$$

### **Theorem 14**

*If the assumptions*$(C_1)-(C_2)$*hold and one of the following conditions is also satisfied:*

$$(D_1)$$
$$\phi _0 \left( \gamma ^2 \int \limits _{\omega }^{1-\omega }{{\mathcal {G}}}_{\alpha} (1,s)ds\right)>1,\ \phi ^{\infty }\sigma _{\alpha}<1\,{{and}}\, \psi _0 \left( \gamma ^2\int \limits _{\omega }^{1-\omega }{{\mathcal {G}}}_{\beta} (1,s)ds\right) >1,\ \psi ^{\infty }\sigma _{\beta} <1.$$*Moreover*, $$\phi _0=\psi _0=\infty$$*and*$$\phi ^\infty =\psi ^\infty =0;$$
$$(D_2)$$
*there exist two constants*$\lambda _1,\lambda _2$*with*$0< \lambda _1\le \lambda _2$*such that*$\phi (t,\cdot ,\cdot )$*and*$\psi (t,\cdot ,\cdot )$*are nondecreasing on*$[0, \lambda _2]$*for all*$t\in [0,1]$,
  $$\begin{aligned} \begin{aligned}&\phi (t, \gamma _\alpha \lambda _1,\gamma _\beta \lambda _1)\ge \frac{\lambda _1}{2} \left( \gamma _\alpha \int \limits _{\omega }^{1-\omega }{{\mathcal {G}}}_\alpha (1,s)ds\right) ^{-1},\\&\psi (t, \gamma _\alpha \lambda _1,\gamma _\beta \lambda _1) \ge \frac{\lambda _1}{2}\left( \gamma _\beta \int \limits _{\omega }^{1-\omega }{{\mathcal {G}}}_\beta (1,s)ds\right) ^{-1}\\&{and}\ \phi (t,\lambda _2,\lambda _2)\le \frac{\lambda _2}{2\sigma _{\alpha }},\ \psi (t,\lambda _2,\lambda _2)\le \frac{\lambda _2}{2\sigma _\beta },\ {for all}\ t\in [0,1]. \end{aligned} \end{aligned}$$

*Then boundary value problem* (1) *has at least one positive solution*.

### *Proof*

As ${{\mathcal {A}}}$ defined in (7) is completely continuous.

**Case I.** Let the conditions of $(D_1)$ hold. Taking $\phi _{0}\left( \gamma ^{2}_{\alpha } \int \limits _{\omega }^{1-\omega }{{\mathcal {G}}}_\alpha (1,s)ds\right) >1,$ then there exists a constant $\epsilon _1>0$ such that

$$\phi (t,u,v)\ge (\phi _{0}-r_1)(u(t)+v(t)),\ \psi (t,u,v) \ge (\psi _{0}-r_2)(u(t)+v(t)), \quad \text {for all}\ t\in [0,1],u,v\in [0,\epsilon _1],$$

where *r*_1_ > 0, and satisfies the conditions

$$(\phi _{0}-r_1)\frac{\gamma ^2_{\alpha }}{2}\int \limits _{\omega }^{1-\omega }{{\mathcal {G}}}_\alpha (1,s)ds\ge 1,\ \ (\psi _{0}-r_1)\frac{\gamma ^2_{\beta }}{2}\int \limits _{\omega }^{1-\omega }{{\mathcal {G}}}_\beta (1,s)ds\ge 1.$$

So for $t\in [0,1], (u,v)\in \partial P_{\epsilon _1},$ we have

$$\begin{aligned} {{\mathcal {A}}}_1(u,v)(t)&=\int \limits _{0}^{1}{{\mathcal {G}}}_\alpha (t,s)\phi (s,u(s),v(s))ds \ge \gamma _{\alpha }\int \limits _{0}^{1}{{\mathcal {G}}}_\alpha (1,s)\phi (s,u(s),v(s))ds\\&\ge (\phi _{0}-r_1)\frac{\gamma _{\alpha }^2}{2}\int \limits _{0}^{1}{{\mathcal {G}}}_\alpha (1,s)ds\Vert (u,v)\Vert \ge \frac{\Vert (u,v)\Vert }{2}. \end{aligned}$$

Analogously

$$\begin{aligned} \begin{aligned} {{\mathcal {A}}}_2(u,v)(t)&=\int \limits _{0}^{1}{{\mathcal {G}}}_\beta (t,s)\psi (s,u(s),v(s))ds\ge \gamma _\beta \int \limits _{0}^{1}{{\mathcal {G}}}_\beta (1,s)\phi (s,u(s),v(s))ds\\&\ge (\psi _{0}-r_2)\frac{\gamma _{\beta }^2}{2}\int \limits _{0}^{1}{{\mathcal {G}}}_\beta (1,s)ds\Vert (u,v)\Vert \ge \frac{\Vert (u,v)\Vert }{2}. \end{aligned}\end{aligned}$$

Therefore, we have

10 $$\begin{aligned} \Vert {{\mathcal {A}}}(u,v)\Vert \ge \Vert {{\mathcal {A}}}_1(u,v)\Vert +\Vert {{\mathcal {A}}}_2(u,v)\Vert \ge \Vert (u,v)\Vert . \end{aligned}$$

Also for $\phi ^\infty \sigma _\alpha <1$ and $\psi ^\infty \sigma _\beta <1,$ there exists a constant say $\bar{\epsilon _2}>0$ such that $\phi (t,u,v)\le (\phi ^\infty +r_2)(u+v),\ \psi (t,u,v)\le (\psi ^\infty +r_2)(u+v),$ for $t\in [0,1], u,v\in (\bar{\epsilon _2},\infty ),$ where $r_2>0$ satisfies the conditions $\sigma _\alpha (\phi ^\infty +r_2)\le 1,\ \sigma _\beta (\psi ^\infty +r_2)\le 1.$ Let $K=\max _{t\in [0,1], u,v\in [0,\bar{\epsilon _2}]}\phi (t,u,v), L=\max _{t\in [0,1], u,v\in [0,\bar{\epsilon _2}]}\psi (t,u,v)$, then $\phi (t,u,v)\le K+(\phi ^\infty +r_2)(u,v), \psi (t,u,v)\le L+(\psi ^\infty +r_2)(u,v).$ Now setting $\max \{\epsilon _1, \bar{\epsilon _2},K\sigma _\alpha (1-\sigma _\alpha (\phi ^\infty +r_2))^{-1}\}\le \frac{\epsilon _2}{2},\max \{\epsilon _1, \bar{\epsilon _2},L\sigma _\beta (1-\sigma _\beta (\psi ^\infty +r_2))^{-1}\}\le \frac{\epsilon _2}{2}.$

So for any $t\in [0,1],(u,v)\in \partial P_{\epsilon _2},$ we obtain

$$\begin{aligned}{{\mathcal {A}}}_1(u,v)(t)&=\int \limits _{0}^{1}{{\mathcal {G}}}_\alpha (t,s)\phi (s,u(s),v(s))ds \le \gamma _{\alpha }\int \limits _{0}^{1}{{\mathcal {G}}}_\alpha (1,s)\phi (s,u(s),v(s))ds\\& \le \int \limits _{0}^{1}{{\mathcal {G}}}_\alpha (1,s)(K+(\phi ^{\infty }+r_2)[u(s)+v(s)]ds\\& \le K\int _0^1{{\mathcal {G}}}_{\alpha }(1,s)ds+(\phi ^\infty +r_2)\int \limits _{0}^{1}{{\mathcal {G}}}_\alpha (1,s)ds\Vert (u,v)\Vert \\&<\frac{\epsilon _2}{2}-\sigma _\alpha (\phi ^\infty +r_2)\frac{\epsilon _2}{2}+(\phi ^\infty +r_2)\sigma _\alpha \Vert (u,v)\Vert < \frac{\epsilon _2}{2}. \end{aligned}$$

Similarly ${{\mathcal {A}}}_2(u,v)(t)<\frac{\epsilon _2}{2}$, as $(u,v)\in \partial P_{\epsilon _2}$, thus, we have

11 $$\begin{aligned} \Vert {{\mathcal {A}}}(u,v)\Vert <\Vert (u,v)\Vert . \end{aligned}$$

**Case II.** If assumptions in $(D_2)$ hold, then inview of definition of *P* for $(u,v)\in \partial P_{\lambda _1}$, we have $\Vert (u,v)\Vert =\lambda _1, \ \text {for}\ t\in J.$ Then from $(D_2)$, we have

$$\begin{aligned}\begin{aligned} {{\mathcal {A}}}_1(u,v)(t)&=\int \limits _{0}^{1}{{\mathcal {G}}}_\alpha (t,s)\phi (s,u(s),v(s))ds \ge \gamma _\alpha \int \limits _{\omega }^{1-\omega }{{\mathcal {G}}}_\alpha (1,s)\phi (s,u(s),v(s))ds\\&\ge \left( \gamma _\alpha \int \limits _{\omega }^{1-\omega }{{\mathcal {G}}}_\alpha (1,s)ds\right) \frac{\lambda _1}{2} \left( \gamma _\alpha \int \limits _{\omega }^{1-\omega }{{\mathcal {G}}}_\alpha (1,s)ds\right) ^{-1}=\frac{\lambda _1}{2}. \end{aligned} \end{aligned}$$

Similarly one can also obtain ${{\mathcal {A}}}_2(u,v)(t)\ge \frac{\lambda _1}{2},$ for $(u,v)\in \partial P_{\lambda _1}$, we get

12 $$\begin{aligned} \Vert {{\mathcal {A}}}(u,v)\Vert =\Vert {{\mathcal {A}}}_1(u,v)\Vert +\Vert {{\mathcal {A}}}_2(u,v)\Vert \ge \Vert (u,v)\Vert . \end{aligned}$$

Also for $(u,v)\in \partial P_{\lambda _2}$, we get that $\Vert (u,v)\Vert =\lambda _2$ for $t\in [0,1]$. Then from $(D_2)$, one can get

$$\begin{aligned} \begin{aligned} {{\mathcal {A}}}_1(u,v)(t)&=\int \limits _{0}^{1}{{\mathcal {G}}}_\alpha (t,s)\phi (s,u(s),v(s))ds \le \int \limits _{0}^{1}{{\mathcal {G}}}_\alpha (1,s)\phi (s,u(s),v(s))ds\\&\le \frac{\lambda _2}{2\sigma _\alpha }\int \limits _{0}^{1}{{\mathcal {G}}}_\alpha (1,s)ds=\frac{\lambda _2}{2}. \end{aligned} \end{aligned}$$

Similarly, one can also obtain that ${{\mathcal {A}}}_2(u,v)(t)\le \frac{\lambda _2}{2}, (u,v)\in \partial P_{\lambda _2}$. Hence, we have

13 $$\begin{aligned} \Vert {{\mathcal {A}}}(u,v)\Vert =\Vert {{\mathcal {A}}}_1(u,v)\Vert +\Vert {{\mathcal {A}}}_2(u,v)\Vert \le \Vert (u,v)\Vert . \end{aligned}$$

Now inview of application of Lemma 5 to (10) and (11) or (12) and (13) implies that ${{\mathcal {A}}}$ has a fixed point $(u_1,v_1)\in \overline{P}_{\epsilon ,\lambda }$ or $(u_1,v_1)\in \bar{P}_{\epsilon _i,\lambda _i}(i=1,2)$ such that $u_1(t)\ge \gamma _\alpha \Vert u_1\Vert >0$ and $v_1(t)\ge \gamma _\beta \Vert v_1\Vert >0, t\in [0,1]$. From which it follows that boundary value problem (1) has at least one positive solution. $\square$

### **Theorem 15**

*Assume that*$(C_1)-(C_3)$*hold. Further the following conditions are also satisfied:*

$$(D_3)$$
*If*$$\phi ^0 \sigma _\alpha <1; \phi ^\infty \left( \gamma ^2_\alpha \int \limits _{\omega }^{1-\omega }{{\mathcal {G}}}_\alpha (1,s)ds\right) >1$$*and*$$\psi ^0 \sigma _\beta <1; \psi ^\infty \left( \gamma ^2_\beta \int \limits _{\omega }^{1-\omega }{{\mathcal {G}}}_\alpha (1,s)ds\right) >1,$$

*Then the boundary value problem* (1) *has at least one positive solutions. Moreover, if*$\phi ^0=\psi ^0=0$*and*$\phi ^\infty =\psi ^\infty =\infty ,$*then the the boundary value problem* (1) *has at least one positive solution*.

### *Proof*

Proof is similar as like the proof of Theorem 14.$\square$

### **Theorem 16**

*Assume that*$(C_1)-(C_3)$*hold. Also the following conditions are also satisfied:*

$$(D_4)$$
*If*$\phi _0\left( \gamma ^2_\alpha \int \limits _{\omega }^{1-\omega }{{\mathcal {G}}}_\alpha (1,s)ds\right)>1, \phi _\infty \left( \gamma ^2_\alpha \int \limits _{\omega }^{1-\omega }{{\mathcal {G}}}_\alpha (1,s)ds\right) >1$*and*$\psi _0\left( \gamma ^2_\beta \int \limits _{\omega }^{1-\omega }{{\mathcal {G}}}_\beta (1,s)ds\right)>1, \psi _\infty \left( \gamma ^2_\beta \int \limits _{\omega }^{1-\omega }{{\mathcal {G}}}_\alpha (1,s)ds\right) >1.$*Moreover*, $\phi _0=\psi _0=\phi _\infty =\psi _\infty =\infty$*is also hold*;
$$(D_5)$$
*there exists*$a>0$*such that*
  $$\max _{t\in [0,1], (u,v)\in \partial P_a}\phi (t,u,v)<\frac{a}{2\sigma _\alpha }$$*and*$$\max _{t\in [0,1], (u,v)\in \partial P_a}\psi (t,u,v)<\frac{a}{2\sigma _\beta }$$.

*Then the boundary value problem* (1) *has at least two positive solutions*$(u,v),(\bar{u},\bar{v})$*such that*

14 $$\begin{aligned} 0<\Vert (u,v)\Vert<a<\Vert (\bar{u},\bar{v})\Vert . \end{aligned}$$

### *Proof*

Let $(D_4)$ hold. Select $\epsilon ,\lambda$ such that $0<\epsilon<\rho <\lambda$. Now if $\phi _0\left( \gamma ^2_\alpha \int \limits _{\omega }^{1-\omega }{{\mathcal {G}}}_\alpha (1,s)ds\right)>1\ \text {and}\ \psi _0\left( \gamma ^2_\beta \int \limits _{\omega }^{1-\omega }{{\mathcal {G}}}_\beta (1,s)ds\right) >1.$

Then like the proof of Theorem 14, we have

15 $$\begin{aligned} \Vert {{\mathcal {A}}}(u,v)\Vert \ge |(u,v)\Vert, \quad \text {for}\ (u,v)\in \partial P_\epsilon . \end{aligned}$$

Now, if $$\phi _\infty \left( \gamma ^2_\alpha \int \limits _{\omega }^{1-\omega }{{\mathcal {G}}}_\alpha (1,s) ds\right)>1\ \text {and}\ \psi _\infty \left( \gamma ^2_\beta \int \limits _{\omega }^{1-\omega }{{\mathcal {G}}}_\beta (1,s)ds\right) >1.$$

Then like the proof of Theorem 14, we have

16 $$\begin{aligned} \Vert {{\mathcal {A}}}(u,v)\Vert \ge \Vert (u,v)\Vert, \quad \text{for}\ (u,v)\in \partial P_\lambda . \end{aligned}$$

Also from $(D_5), (u,v)\in \partial P_\rho ,$ we get

$$\begin{aligned} \begin{aligned} {{\mathcal {A}}}_1(u,v)(t)&=\int _0^1 {{\mathcal {G}}}_\alpha (t,s)\phi (s,u(s),v(s))ds\\&\le \int _0^1 {{\mathcal {G}}}_\alpha (1,s)\phi (s,u(s),v(s))ds<\frac{\rho }{2\sigma _\alpha }\int _0^1 {{\mathcal {G}}}_\alpha (1,s)ds=\frac{\rho }{2}. \end{aligned} \end{aligned}$$

Similarly, we have ${{\mathcal {A}}}_1(u,v)(t)< \frac{\rho }{2}$ as $(u,v)\in \partial P_\rho$. Hence, we have

17 $$\begin{aligned} \Vert {{\mathcal {A}}}(u,v)\Vert < |(u,v)\Vert, \quad \text {for}\ (u,v)\in \partial P_\rho . \end{aligned}$$

Now by the use of Lemma 5 to (15) to (17) gives that ${{\mathcal {A}}}$ has a fixed point $(u,v)\in \partial \overline{P}_{\epsilon ,\rho }$ and a fixed point in $(\bar{u},\bar{v})\in \partial \overline{P}_{\rho ,\lambda }.$ Hence it implies that the boundary value problem (1) has at least two positive solutions $(u,v), (\bar{u},\bar{v})$ such that $\Vert (u,v)\Vert \ne \rho$ and $\Vert (\bar{u},\bar{v})\Vert \ne \rho$. Thus relation (14) holds. The proof is completed. $\square$

### **Theorem 17**

*Assume that*$(C_1)-(C_3)$*hold and also the following conditions are satisfied:*

$$(D_6)$$
$$\sigma _\alpha \phi _{0}<1$$*and*$$\phi _\infty \sigma _\alpha<1;\ \sigma _\beta \psi _{0}<1,$$*and*$$\psi _\infty \sigma _\beta <1;$$
$$(D_7)$$
*there exist*$\rho >0$*such that*
  $$\begin{aligned}&\max _{t\in J, (u,v)\in \partial P_\rho }\phi (t,u,v)>\frac{\rho }{2}\left( \gamma ^2_{\alpha } \int \limits _{\omega }^{1-\omega }{{\mathcal {G}}}_\alpha (1,s)ds\right) ^{-1},\\&\max _{t\in J, (u,v)\in \partial P_\rho }\psi (t,u,v) >\frac{\rho }{2}\left( \gamma ^2_{\beta } \int \limits _{\omega }^{1-\omega }{{\mathcal {G}}}_\beta (1,s)ds\right) ^{-1} \end{aligned}$$
  *such that*
  $$\begin{aligned} 0<\Vert (u,v)\Vert<\rho <\Vert (\bar{u},\bar{v})\Vert . \end{aligned}$$

*Then the boundary value problem* (1) *has at least two positive solutions.*

### *Proof*

Proof is like the proof of Theorem 16. $\square$

Similarly for multiplicity the following theorems can be easily deduced:

### **Theorem 18**

*Let*$(C_1)-(C_3)$*hold. If there exist* 2*mpositive numbers*$\mathbf {w}_k,\hat{\mathbf {w}}_k,\;k=1,2\ldots m$*with*$\mathbf {w}_1<\gamma _\alpha \hat{\mathbf {w}}_1< \hat{\mathbf {w}}_1< \mathbf {w}_2< \gamma _\alpha \hat{\mathbf {w}}_2< \hat{\mathbf {w}}_2 \ldots \mathbf {w}_m<\gamma _\alpha \hat{\mathbf {w}}_m < \hat{\mathbf {w}}_m$*and*$\mathbf {w}_1<\gamma _\beta \hat{\mathbf {w}}_1< \hat{\mathbf {w}}_1< \mathbf {w}_2< \gamma _\beta \hat{\mathbf {w}}_2< \hat{\mathbf {w}}_2 \ldots \mathbf {w}_m<\gamma _\beta \hat{\mathbf {w}}_m < \hat{\mathbf {w}}_m$*such that*

$$(D_{8})$$
$$\phi (t, u(t), v(t)) \ge \mathbf {w}_k \left( \gamma _\alpha \int \limits _0^1{{\mathcal {G}}}_\alpha (1,s)ds\right) ^{-1},$$*for*$$(t,u,v)\in [0,1]\times [\gamma _\alpha \mathbf {w}_k,\mathbf {w}_k]\times [\gamma _\beta \mathbf {w}_k,\mathbf {w}_k],$$*and*
  $$\begin{aligned} \phi (t, u(t), v(t)) \le \sigma ^{-1}_{\alpha }\hat{\mathbf {w}}_k, \text {for}\ (t,u,v)\in [0,1]\times [\gamma _\alpha \hat{\mathbf {w}}_k, \hat{\mathbf {w}}_k]\times [\gamma _\beta \mathbf {w}_k,\mathbf {w}_k], k=1,2\ldots m, \end{aligned}$$
$$(D_{9})$$
$$\psi (t, u(t), v(t)) \ge \mathbf {w}_k \left( \gamma _\beta \int \limits _0^1{{\mathcal {G}}}_\beta (1,s)ds)\right) ^{-1}, for (t,u,v)\in [0,1]\times [\gamma _\beta \mathbf {w}_k,\mathbf {w}_k]\times [\gamma _\alpha \mathbf {w}_k,\mathbf {w}_k], and \psi (t, u(t), v(t)) \le \sigma _{\beta }^{-1} \hat{\mathbf {w}}_k, \text {for} \ (t,u,v)\in [0,1]\times [\gamma _\alpha \mathbf {w}_k,\mathbf {w}_k]\times [\gamma _\beta \hat{\mathbf {w}}_k, \hat{\mathbf {w}}_k],\; k = 1,2\ldots m.$$

*Then the boundary value problem* (1) *has at leastm*-*positive solutions*$(u_k, v_k),$*satisfying*

$$\begin{aligned} \mathbf {w}_k \le \Vert (u_k, v_k)\Vert \le \hat{\mathbf {w}}_k,\; k=1,2\ldots m. \end{aligned}$$

### **Theorem 19**

*Suppose that*$(C_1)-(C_3)$*holds. If there exist* 2*mpositive numbers*$\mathbf {w}_k,\hat{\mathbf {w}}_k,\;k=1,2\ldots m$, *with*$\mathbf {w}_1< \hat{\mathbf {w}}_1< \mathbf {w}_2< \hat{\mathbf {w}}_2 \ldots< \mathbf {w}_m < \hat{\mathbf {w}}_m$*such that*

$$(D_{10})$$
$\phi$*and*$\psi$*are non-decreasing on*$[0,\hat{\mathbf {w}}_m]$*for all*$t\in [0,1];$
$$(D_{11})$$
$$\phi (t, u(t), v(t))\ge \mathbf {w}_k \left( \gamma _\alpha \int \limits _{\omega }^{1-\omega } {{\mathcal {G}}}_\alpha (1,s)ds)\right) ^{-1},\ \phi (t, u(t), v(t))\le \dfrac{\hat{\mathbf {w}}_k}{\sigma _\alpha }, \;k=1,2\ldots m, \psi (t, u(t), v(t))\ge \mathbf {w}_k \left( \gamma _\beta \int \limits _{\omega }^{1-\omega } {{\mathcal {G}}}_\beta (1,s)ds)\right) ^{-1},\ \psi (t, u(t), v(t))\le \dfrac{\hat{\mathbf {w}}_k}{\sigma _\beta }, \;k=1,2\ldots m.$$

*Then the boundary value problem* (1) *has at leastm*-*positive solutions*$(u_k,v_k),$*satisfying*

$$\begin{aligned} \mathbf {w}_k \le \Vert (u_k,v_k)\Vert \le \hat{\mathbf {w}}_k,\;v=1,2\ldots m. \end{aligned}$$

## Examples

We conclude the paper with the following examples.

### *Example 20*

Consider the coupled system as follow

18 $$\begin{aligned} \left\{ \begin{aligned}&D^{\frac{5}{2}}u(t) + \dfrac{t+1}{ 4}[\Gamma \big (\frac{5}{2}\big )|u(t)|+\cos |v(t)|]=0,\ t\in [0,1], u, v\ge 0, \\ &D^{\frac{5}{2}}v(t) + \dfrac{t^2+1}{ 4}[\sin |u(t)|+v(t)]=0,\ t\in [0,1], u, v\ge 0, \\&u^{(i)}(0) =v^{(i)}(0)=0,\ i=0,1\\&D^{\frac{1}{2}}u(1) = \frac{1}{2}D^{\frac{1}{2}}u\left( \frac{1}{2}\right) ,\ D^{\frac{1}{2}}v(1) =\frac{1}{2} D^{\frac{1}{2}}v\left( \frac{1}{2}\right) . \end{aligned}\right. \end{aligned}$$

Since $\phi (t,u(t),v(t)) =\dfrac{t+1}{ 4}[\Gamma (\frac{5}{2})|u(t)|+\cos |v(t)|] ,\quad \psi (t, u(t),v(t))=\dfrac{t^2+1}{ 4}[\sin |u(t)|+|v(t)|].$ Also as $0<\gamma , \delta <n-2, n=[2.5]+1=3$ and $\gamma =\delta =\frac{1}{2}.$ Then

$$\begin{aligned} \begin{aligned}&|\phi (t,u_2,v_2)-\phi (t,u_1,v_1)| \le \Gamma (\frac{5}{2})\dfrac{t+1}{ 4}|u_2-u_1|+\dfrac{t+1}{ 4}|v_2-v_1|,\\&|\psi (t,u_2,v_2)-\psi (t,u_1,v_1)| \le \dfrac{t^2+1}{ 4}|u_2-u_1|+\dfrac{t^2+1}{4}|v_2-v_1|. \end{aligned} \end{aligned}$$

where $f_1(t)=\frac{t+1}{4}\Gamma (\frac{5}{2}),g_1(t)= \frac{t+1}{4},\ f_2(t)=g_2(t)=\dfrac{t^2+1}{4},$ from which we have

$$\begin{aligned} A=\left[ \begin{aligned}&\int \limits _{0}^{1} {{\mathcal {G}}}_\alpha (1,s)f_1(s)ds)&\int \limits _{0}^{1} {{\mathcal {G}}}_\alpha (1,s)g_1(s(ds) \\&\int \limits _{0}^{1} {{\mathcal {G}}}_\beta (1,s)f_2(s)ds)&\int \limits _{0}^{1} {{\mathcal {G}}}_\beta (1,s)g_2(s)ds) \end{aligned} \right] =\left[ \begin{aligned}&\frac{5}{36}&\frac{5}{27\sqrt{\pi }} \\&\frac{22}{135\sqrt{\pi }}&\frac{22}{135\sqrt{\pi }} \end{aligned} \right] . \end{aligned}$$

By standard calculation, we can obtain that $\rho (A)=0.2479<1,$ hence by the use of Theorem 13 boundary value problem (18) has a unique positive solution.

### *Example 21*

Consider the system of non-linear fractional differential equations.

19 $$\begin{aligned} \left\{ \begin{aligned}&D^{\frac{7}{3}} u(t) + a(t)\sqrt{u(t)+v(t)} = 0, \;D^{\frac{5}{2}} v(t) + b(t)\root 3 \of {u(t)+v(t)}=0,\quad t\in (0,1),\\&u(0) = u^{\prime }(0)=0,\ v(0)=v^{\prime }(0)=0,\\&\ D^{\frac{1}{3}} u(1) = \frac{1}{3}D^{\frac{1}{3}} u\left( \frac{1}{2}\right) ,\ \ D^{\frac{1}{2}} v(1) = \frac{1}{2}D^{\frac{1}{2}}v\left( \frac{1}{2}\right) , \end{aligned}\right. \end{aligned}$$

where $\phi (t,u,v)=a(t)\sqrt{u(t)+v(t)}$ and $\psi (t,u,v)=b(t)\root 3 \of {u(t)+v(t)},\ \delta =\frac{1}{3},\ \gamma =\frac{1}{2}.$ and $a(t),\ b(t):[0,1]\rightarrow [0,\infty )$ are continuous.

Now $\phi ^0 = \lim \limits _{(u,v)\rightarrow 0} \dfrac{\phi (t,u,v)}{u+v} = \infty$, similarly $\psi ^0=\infty .$ Also by simple calculation we can get that $\phi ^{\infty }=0 = \psi ^{\infty }.$ Thus by Theorem 14, boundary value problem (19) has at least one positive solution.

### *Example 22*

Consider the following boundary value problem

20 $$\begin{aligned} \left\{ \begin{aligned}&D^{\frac{9}{2}}u(t)+(1-t^2)[u(t)+v(t)]^{2} = 0,\ D^{\frac{14}{3}}v(t)+[u(t)+v(t)]^{3} = 0,\quad t \in (0,1),\\&u^{(i)}(0)=v^{(i)}=0, \quad i=0,1,2,3,\\&D^{\frac{5}{2}}u(1)=\frac{1}{4}D^{\frac{5}{2}}u\left( \frac{1}{4}\right) ,\ D^{\frac{5}{2}}v(1)=\frac{1}{3}D^{\frac{5}{2}}v\left( \frac{1}{3}\right) . \end{aligned}\right. \end{aligned}$$

from (20), we have $\gamma =\delta =\frac{5}{2},$ as $n=5, 0<\gamma , \delta <3.$ By simple calculation we obtain that $\phi ^0=\psi ^0=0$ and $\phi ^\infty =\psi ^\infty =\infty .$ Thus by Theorem 15, the boundary value problem (20) has a positive solution.

### *Example 23*

Consider the following boundary value problem

21 $$\begin{aligned} \left\{ \begin{aligned}&D^{\frac{9}{2}}u(t)+\frac{(1+t^2)[u^2(t)+v(t)]}{(4t^2+4)\sigma _\alpha } = 0,\quad t\in (0,1),\\&D^{\frac{14}{3}}v(t)+\frac{(t^3+1)[u(t)+v^2(t)]}{(4t^3+4)\sigma _\beta } = 0, \quad t\in (0,1),\\&u^{(i)}(0)=v^{(i)}=0, \quad i=0,1,2,3,\\&D^{\frac{5}{2}}u(1)=\frac{1}{4}D^{\frac{5}{2}}u\left( \frac{1}{4}\right) ,\ D^{\frac{5}{2}}v(1)=\frac{1}{3}D^{\frac{5}{2}}v\left( \frac{1}{3}\right) . \end{aligned}\right. \end{aligned}$$

where $\gamma =\delta =\frac{5}{2},$ as $n=5, 0<\gamma , \delta <3.$ Also, by simple calculation we obtain that $\phi _0=\psi _0=\infty$ and $\phi _\infty =\psi _\infty =\infty .$

Further for all $(t,u,v) \in [0,1]\times [0,1]\times [0,1],$ we have

$$\begin{aligned} \begin{aligned}&\phi (t,u,v) \le \frac{(t^2+1)2}{4(t^2+1)\sigma _\alpha }=\frac{\sigma ^{-1}_\alpha }{2},\ \psi (t,u,v) \le \frac{(t^3+1)2}{4(t^3+1)\sigma _\beta }=\frac{\sigma ^{-1}_\beta }{2}. \end{aligned} \end{aligned}$$

Thus all the assumptions of Theorem 16 are satisfied and also $a=1.$ Hence by Theorem 16, the boundary value problem (21) has at least two positive solutions $(u_1,v_1)$ and $(u_2,v_2)$ which satisfy

$$\begin{aligned} 0<\Vert (u_1,v_1)\Vert<1<\Vert (u_2,v_2)\Vert . \end{aligned}$$

## Non-existence of positive solution

In this section, we discuss the non-existence of positive solution to the coupled system (1) of fractional order differential equations.

### **Theorem 24**

*Assume that*$(C_1)-(C_3)$*hold and*$\phi (t,u,v) < \frac{\Vert (u,v)\Vert }{2\sigma _\alpha }$*and*$\psi (t,u,v)<\frac{\Vert (u,v)\Vert }{2\sigma _\beta }$*for all*$t\in [0,1],u>0,v>0,$*then the boundary value problem* (1) *has no positive solution*.

### *Proof*

On contrary let (*u*, *v*) be the positive solution of boundary value problem (1) . Then $(u,v)\in P$ for $0<t<1$ and

$$\begin{aligned}\Vert (u,v)\Vert &= \Vert u\Vert +\Vert v\Vert =\max _{t\in [0,1]}|u(t)|+\max _{t\in [0,1]}|v(t)|\\& \le \max _{t\in [0,1]} \int \limits _{0}^{1} {{\mathcal {G}}}_\alpha (t,s)|\phi (s,u(s),v(s))|ds+\max _{t\in [0,1]} \int \limits _0^1 G_\beta (t,s)|\psi (s,u(s),v(s)|ds\\&\quad< \int \limits _0^1G_\alpha (1,s)\frac{\Vert (u,v)\Vert }{2\sigma _\alpha }ds+\int \limits _0^1G_\beta (1,s)\frac{\Vert (u,v)\Vert }{2\sigma _\beta }ds\\& \Rightarrow \Vert (u,v)\Vert < \Vert (u,v)\Vert , \end{aligned}$$

which is contradiction. So boundary value problem (1) has no positive solution. Hence proof is completed. $\square$

### **Theorem 25**

*Let*$$(C_1)-(C_3)$$*holds and if*$$\phi (t,u(t),v(t))>\frac{\Vert (u, v)\Vert }{2}\left( \gamma _\alpha ^2 \int \limits _{\omega }^{1-\omega }G_\alpha (1,s)ds\right) ^{-1},$$$$\psi (t,u(t),v(t))> \frac{\Vert (u, v)\Vert }{2}\left( \gamma _\beta ^2 \int \limits _{\omega }^{1-\omega }G_\beta (1,s)ds\right) ^{-1}$$*for all*$$t\in [0,1], u>0$$*and*$$v>0$$. *Then boundary value problem* (1) *has no positive solution*.

### *Proof*

Proof is just like the proof of Theorem 24, so we omit it.$\square$

### *Example 26*

In this section we provide an example which illustrate the results of Theorems 24 and 25 respectively. Consider the system of non linear fractional differential equations:

22 $$\begin{aligned} \left\{ \begin{aligned}&D^{\frac{5}{2}}u(t)+\dfrac{(2u^2(t)+u(t))(20 + \cos v(t))}{u(t)+v(t)+1} = 0, \quad t\in [0,1],\\&D^{\frac{5}{2}}v(t)+\dfrac{(2v^2(t)+v(t))(20 + \cos v(t))}{u(t)+v(t)+1} = 0, \quad t\in [0,1],\\&u^{(i)}(0) = v^{(i)}(0)=0,\ \quad i=0,1,\\&D^{\frac{1}{2}}u(1)=\frac{1}{10}D^{\frac{1}{2}}u\left( \frac{1}{2}\right) D^{\frac{1}{2}}v(1)=\frac{1}{10}D^{\frac{1}{2}}v\left( \frac{1}{2}\right) . \end{aligned}\right. \end{aligned}$$

Since $(C_1)-(C_3)$ holds and also $\gamma =\delta =\frac{1}{2},$ as $n=3, 0<\gamma , \delta <1$.

$$\begin{aligned} \phi ^0=\psi ^0=20,\ \ \phi ^\infty =\psi ^\infty =43,\ \ 20\Vert (u,v)\Vert<\phi (t,u(t),v(t))< 43\Vert (u,v)\Vert \end{aligned}$$

$$\begin{aligned} 20\Vert (u,v)\Vert<\psi (t,u(t),v(t))< 43\Vert (u,v)\Vert \ \hbox {and}\ \phi (t,u(t),v(t))< 43\Vert (u,v)\Vert < \dfrac{\Vert (u,v)\Vert }{\sigma _\alpha }, \end{aligned}$$

where $$\sigma _\alpha \approx 0.876126$$ and $$\sigma _\beta \approx .475675$$

**Case I:** Now $\phi (t,u(t),v(t))< \dfrac{\Vert (u,v)\Vert }{\sigma _\alpha }\approx 1.1413\Vert (u,v)\Vert$ implies that $\phi (t,u(t),v(t))<43\Vert (u,v)\Vert \approx 1.1413\Vert (u,v)\Vert$ and $\psi (t,u(t),v(t))<43\Vert (u,v)\Vert \approx 2.1022\Vert (u,v)\Vert .$ Thus by Theorem 24, boundary value problem (22) has no positive solution.

**Case II:** Also $\phi (t,u(t),v(t))>20\Vert (u,v)\Vert >\Vert (u,v)\Vert \left( \gamma _\alpha ^2 \int \limits _{\frac{1}{3}}^{\frac{2}{3}}{{\mathcal {G}}}_\alpha (1,s)ds\right) ^{-1} \approx 15.8688\Vert (u,v)\Vert$ and $\psi (t,u(t),v(t))>2\Vert (u,v)\Vert >\Vert (u,v)\Vert \left( \gamma _\beta ^2 \int \limits _{\frac{1}{3}}^{\frac{2}{3}}{{\mathcal {G}}}_\beta (s,s)ds\right) ^{-1} \approx 12.678\Vert (u,v)\Vert .$

Then by Theorem 25, boundary value problem (22) has no solution.

## Conclusion

In this article, we have developed sufficient conditions for the multiplicity results of positive solutions to a highly nonlinear coupled system of fractional order differential equations. Our paper is the generalization of the work carried out in Goodrich (2010), Jalili and Samet (2014), Rehman and Khan (2010). In Jalili and Samet (2014), the authors studied the concerned coupled system with homogenous boundary conditions involving fractional order derivative, but we extended this work with taking non-homogenous boundary condition involved fractional order derivative of Riemann- Liouville type. By using classical fixed point theorems, we have successfully developed conditions under which the considered coupled system has multiple solutions. Moreover, uniqueness and non existence results have also been established . Numerous examples have been provided which justify the results developed by us.
